# The Economic Impacts of Chronic Illness on Households of Patients in Ile-Ife, South-Western Nigeria

**DOI:** 10.7759/cureus.1756

**Published:** 2017-10-07

**Authors:** Paul T Okediji, Adedolapo O Ojo, Akinwumi I Ojo, Ademola S Ojo, Opeyemi E Ojo, Emmanuel A Abioye-Kuteyi

**Affiliations:** 1 Research & Development, Sebeccly Cancer Center, Lagos; 2 College of Health Sciences, Obafemi Awolowo University, Ile-Ife, Nigeria; 3 Department of Surgery, Obafemi Awolowo University, Ile-Ife, Nigeria; 4 Department of Medicine, Ekiti State University Teaching Hospital, Ekiti State. Nigeria; 5 Department of Family Medicine, Obafemi Awolowo University, Ile-Ife, Nigeria

**Keywords:** nigeria, lmic, chronic illness, economic impacts, cost of illness, direct costs, coping, household

## Abstract

Introduction

Chronic illnesses are slowly becoming more prevalent worldwide. The implications and ramifications of these illnesses vary and affect not only the patient but the entire household in many ways. This research focuses on the economic implications of this category of illnesses on the entire household. The aim is to determine the economic implications of chronic illnesses on households of patients in selected health facilities in Ile-Ife, Nigeria, and to elucidate the various coping strategies applied by households in low and middle income countries (LMICs) to keep up with these economic implications.

Methods

This study features a descriptive cross-sectional survey design with a total sample of 443. The target population consists of individuals with chronic diseases in selected health care facilities in Ile-Ife.

Results

The mean household monthly incomes before and after illness episodes were found to be $335.84 and $318.01, respectively. The mean direct cost of chronic illness was $137.72 with about 79% (n=350) of the respondents spending more than 10% of the monthly household income on health. The indirect costs of illness were a loss of productivity of 18.9% and 5.1% for patients and caregivers, respectively. A large percentage of the respondents resorted to borrowing (44.7%; n=198), while another 5.0% (n=22) sold assets, and 8.6% (n=38) had access to health insurance in order to cope with the economic impacts of the illness.

Conclusion

The study showed that chronic illnesses imposed high and catastrophic cost burdens on patients and their households. The lack of effective coping strategies points at the need for policymakers to improve access to specialized care and increase coverage of formal health insurance so as to ameliorate the significant economic impacts that chronic illnesses have on entire households.

## Introduction

Chronic illnesses such as diabetes mellitus, cardiovascular disease, cerebrovascular disease, cancers, chronic respiratory diseases, mental illnesses, and other non-communicable diseases have become major issues globally [1]. Between 1980 and 2015, non-communicable chronic illnesses accounted for 71.3% (about 40 million) of all deaths [2]. Furthermore, the World Health Organization (WHO) estimated that 80% of the deaths due to chronic illnesses occurred in low and middle-income countries (LMICs) with developing economies [3]. These chronic and slowly progressive illnesses are becoming increasingly prevalent as a result of significant changes in lifestyle, reduction in death rates, increased longevity, and an improvement in health services in many countries. Attendant implications include a higher medical bill which can lead to economic impoverishment (which is broadly defined as a process of household income and asset depletion which cause consumption levels to fall below minimum needs) and significant morbidity and mortality in low and middle-income countries (LMICs), including Nigeria [4].

Chronic illnesses have been associated with disability and this can have damaging, or serious economic implications on the individual and his/her family as they generally deprive individuals of their productivity and health potentials [5]. From the national perspective, chronic illnesses reduce both work capacity and life expectancy, and therefore, economic productivity; leading to a reduction in the quantity and quality of the nation’s labor force [6]. Invariably, the burden of chronic illnesses primarily impacts the income and depletes investment savings of the individual and the household. Usually, the direct economic impacts on the household may arise typically in the form of hospital bills, caregiver allowances, nursing home bills, and other aspects of care [5]. For LMICs which place poverty reduction at the center of developmental targets and strategies, the increase in the prevalence of chronic illnesses poses a serious threat to the achievement of these targets.

Different approaches have been employed in studying the economic burden of illnesses such as the cost of illness (COI), microeconomic and macroeconomic approaches [5]. The COI approach is suitable for this study because of its ability to highlight the economic magnitude of a chronic disease and/or its associated risk factors, which accounts for both direct medical and non-medical costs, and indirect losses due to foregone productivity [5, 7]. It is clear that there are costs associated with being sick – the cost of obtaining treatment for the chronic illness, the cost of travel to access care, the income forgone because of the inability to work or perform normal duties as a result of the illness. These costs have been divided into direct, indirect, and intangible costs [7].

Direct costs estimate the household’s spending on medical care as far as the prevention, diagnosis, and treatment of the illness are concerned [5]. These include the costs accrued as a result of the use of medications, hospital admissions, ambulances or other means of transportation to the hospital or point-of-care, in-patient care or out-patient care (clinics), rehabilitation, and community health services. On the other hand, indirect costs represent the loss of man hours or labor that is caused by morbidity/mortality arising from the disease. The human capital approach of measuring indirect costs looks at the lost labor time due to the illness which often translates to a reduced household capacity to earn income, especially at a time when it needs the additional money to pay for treatment [8, 9]. There is also the much broader “willingness-to-pay” method of measuring indirect costs, which focuses on the amount of money people are willing to pay for relatively small changes to avert the risk of death [5]. The third category, and most difficult to measure, is the intangible cost, which encompasses the psychological dimensions that people attribute to illness, usually in the form of pain, anxiety, suffering, and bereavement.

The household has been selected in similar studies as the preferred unit of analysis for evaluating illness costs because: (i) major decisions concerning treatment and coping strategies are determined at the household level, (ii) most of the costs of managing an illness are borne by both the sick individual and other members of the household, and (iii) the costs of illness fall on the household budget [8]. The “cost burden” has to do with the proportion of the family income spent on obtaining treatment; and when this goes beyond what the family can easily bear, it triggers the use of coping strategies such as borrowing or selling off of assets. For many Nigerian families, the first point of call for seeking for funds is from social networks (the larger/extended family, friends, social groups), or local organizations that offer credit. Both illness costs and coping strategies have serious implications for household asset portfolios and processes of impoverishment [8]. There is presently a dearth of information on the economic impact of chronic illnesses on households in the Nigerian population. Hence, the primary focus of this study is to assess the effects of chronic illnesses on the expenditure of the household and labor income; and to investigate the influence of health insurance and other sources of funds on the economic shocks of chronic diseases.

## Materials and methods

A total of 443 patients who were either attending clinics or on admission for various chronic illnesses at the Obafemi Awolowo University Teaching Hospitals’ Complex (OAUTHC), and the Seventh-Day Adventist Hospital; both in Ile-Ife, Nigeria, between February and March 2012 were sampled. Patients were consecutively recruited, and included in this research if: (i) they were 18 years and above, (ii) had a definitive diagnosis of at least one chronic illness, and (iii) had been in treatment for their disease for at least three months. The length of time was given for them to have experienced significant costs associated with managing their condition. Patients who were too ill to respond and/or did not give informed consent were excluded from the study.

Data was obtained from the patients with the aid of an interviewer-administered questionnaire adapted from the study by Jeon, et al. [10]. The questionnaire sought information on socio-demographic data, self-reported health status, treatment behavior, economic status, affordability of health care, affordability of other essentials, and coping strategies. Information was also obtained on the patients’ current work status, loss of productive labor time due to illness, presence of financial difficulties or financial pressure, actual income lost for each respondent, actual cost of the patient’s healthcare per week or per month, effects on the caregiver, and whether the respondents received any government benefits. Questionnaire information was entered into a microcomputer and analyzed with the level of statistical significance set at < 0.05. Ethical approval was obtained from the Ethics and Research Committee of the OAUTHC, Ile-Ife. All respondents gave informed consent prior to their participation in the study, and the study was conducted in line with the principles of the Helsinki declaration.

## Results

The results obtained were analyzed with the use of the Statistical Package for the Social Sciences [SPSS] (IBM Corp., Armonk, NY) version 16 software. Descriptive and inferential statistics (chi-square analysis and independent t-tests) were performed and the results are presented in tables. 

Subjects’ characteristics

A large proportion (64.8%; n=287) of the respondents were between 18 and 64 years (Table 1). There were more females (52.1%; n=231) than males (47.9%; n=212). Concerning employment, 64.6% (n=286) of the respondents were in a paid employment while the rest were not in any form of paid employment, and this includes those who were retired, unemployed, or not active. Patients were asked about their self-evaluated health status and about 71% (n=315) said that their health was at least good while 2.9% (n=13) had a perception of poor health. Hypertension was found to be the most common chronic illness among the population studied (65.2%; n=289), followed by diabetes (32.1%; n=142). About 37% (n=164) had illnesses lasting between three and 12 months, 31.8% (n=141) had been ill for 13-60 months, while the remaining 31.2% (n=138) had been ill for more than five years. The first place of treatment was the health care facility for about 92% (n=407) of subjects.

**Table 1:** Socio-demographic, clinical and behavioral characteristics of subjects

	Frequency (n=443)	Percentage (%)
Age		
18 - 64 years	287	64.8
65 years and older	156	35.2
Gender		
Male	212	47.9
Female	231	52.1
Marital status		
With a partner (married/co-habiting)	341	77.0
No partner (single/separated/divorced/widowed)	102	23.0
Employment status		
Paid Employment/self employed	286	64.6
Not in paid employment	157	35.4
Had a Carer	378	85.3
Type of chronic illness*		
Hypertension	289	65.2
Diabetes	142	32.1
Cancer (all types)	14	3.2
Bone or joint problems	37	8.4
Chronic kidney disease	7	1.6
Chronic liver disease	23	5.2
Heart disease	32	7.2
Mental illness	48	10.8
Others	8	1.8
Duration of illness		
<12 months	164	37.0
13 – 60 months	141	31.8
>60 months	138	31.2
Place of first treatment before going to the hospital		
Pastor	8	1.8
Family members	7	1.6
Chemist	18	4.1
Traditional healer	3	0.7
Healthcare facility	407	91.9
Drug adherence over the last 30 days	148	33.4
Use of alternative treatments like herbal remedies	107	24.2
*Some of the respondents had more than one of the index conditions.		

Direct costs of chronic illness to households

The mean direct cost of chronic illness obtained from the respondents was $137.72, constituting 43.3% of the mean household income (Table 2). The mean monthly household spending on non-health goods and services was $180.30. When the direct cost of treatment of the subject’s chronic illnesses was expressed as a proportion of the monthly household income, only 21.9% of the respondents spent less than 10% of their monthly income on health while more than 40% of subjects spent over 40% of monthly income on healthcare (Table 3). A comparison of the means of the household monthly income, before and after the onset of illness, showed no statistical difference (p=0.07). Medications constituted the bulk of the direct costs, making up 49.7% of the whole household spending on health. Transportation cost was the least, taking up 6.9% of the direct costs.

**Table 2:** Household monthly income and direct costs of chronic illness among respondents

	n = 443	
Monthly Income	Mean ($)	SD ($)
Household monthly income before the illness	335.84	602.95
Household monthly income since the illness started	318.01	533.43
Change in monthly income	-17.82	208.53
Direct average monthly costs of chronic illness		
Drugs	68.43	177.97
Laboratory investigations	18.62	70.82
Admission	28.92	153.31
Other forms of treatment (rehabilitation, community health services)	12.94	82.68
Transportation to the hospital	8.81	28.47
Total	137.72	359.54
Household monthly non-health expenditure	180.30	647.41

 

**Table 3:** Household monthly income; and monthly direct health expenditure as a proportion of household monthly income of respondents

	Frequency (n=443)	Percentage (%)
Monthly income		
≤ $138	206	46.5
>$138	237	53.5
Direct chronic illness costs		
≤ 10%	97	21.9
11% - 20%	76	17.1
21% - 30%	55	12.4
31% - 40%	26	5.9
> 40%	189	42.7

Indirect costs of chronic illness to households

The indirect costs of the chronic illness on both patients and caregivers who were members of the households were estimated using the number of work days lost per month to illnesses and change of jobs during the period of the index illness. As shown in Table 4, on the average, 5.3 work days were lost by patients in a month, constituting about 18.9% lost productivity while caregivers only lost on average 1.4 work days constituting 5.1% lost productivity. About 15% of subjects lost more than 50% of work days in the preceding month. Also, approximately 12% of caregivers had to seek other ways to make more money to take care of family needs as a result of the burden of the illness.

**Table 4:** Indirect costs of illness as days lost within the last 30 days to patients and caregivers SD: standard deviation

	n=443	
Days lost per patient per month	Frequency	Percent (%)
< 5 days	353	79.7
6-10 days	10	2.3
11-15 days	15	3.4
> 15 days	65	14.7
Indirect costs	Mean (days)	SD (days)
Patient	5.30	10.37
Caregiver	1.42	5.53

Affordability of healthcare

Considering the impact of the costs of healthcare, 38.6% (n=171) of the respondents had at least one unsatisfied basic need such as food, shelter, and clothing partly due to the burden of chronic illness (Table 5). A significant proportion of subjects could not afford key elements of care for their condition, as shown in Table 5, while between 35 and 50% of subjects had difficulties securing basic necessities, maintaining lifestyle and social integration due to the economic burden of their chronic illness. In particular, 35.2% (n=156) of the respondents experienced financial difficulties in the area of economic capabilities. These financial difficulties influenced their treatment choices, as money was the determining factor in 8.8% (n=39) of treatment choices, although the doctor’s prescription (88.5%; n=392) and family’s decision (2.7%; n=12) influenced treatment choices significantly. Thus, most respondents made health care their priority need and obtained health care at the expense of other needs.

**Table 5:** Affordability of healthcare and other essentials among respondents

Description	Frequency	Percentage
Ability to finance all medications	268	60.5%
Ability to finance all laboratory investigations	316	71.3%
Ability to finance admission when necessary	249	56.2%
Difficulty purchasing basic necessities since the illness started	156	35.2%
Difficulty in maintaining normal lifestyle since the illness started	207	46.7%
Had to cut back on some things usually bought before the illness	210	47.4%
Had reduced participation in some social and leisure activities due to cost of treatment	196	44.2%

Coping strategies among respondents

It was observed that 44.7% (n=198) of the respondents had, at one time or the other, borrowed money in order to meet the costs of treatment (Table 6). Of the lenders, friends constituted the majority (54.5%; n=108), followed by family (19.2%; n=38), cooperative societies (18.2%; n=36), church (4.5%; n=9), and banks (3.5%; n=7). About 5.0% (n=22) of the respondents had to sell assets to finance treatment. Of the 443 respondents, only 8.6% (n=38) had health insurance. Most of the insured respondents (94.7%; n=36) claimed that the health insurance reduced the impact of the chronic illness on the finances of the household.

**Table 6:** Coping strategies in use among respondents

	Frequency (%)
Had borrowed money for any aspect of treatment at any time	198 (44.7%)
Source of borrowed money (n=198)	
Bank	7 (3.5%)
Cooperative society	36 (18.2%)
Church	9 (4.5%)
Friends	108 (54.5%)
Family	38 (19.2%)
Sold assets to get money for treatment	22 (5.0%)
Health insurance ownership	38 (8.6%)
Health insurance reduced the impact of the chronic illness on the household	36 (94.7%)

The majority of respondents with a single chronic illness spent less than 10% of their average household monthly income on health, while the majority of those who had multiple chronic illnesses spent more than 10% (p=0.001; Table 7). There was no statistically significant difference in the number of days lost to illness (indirect costs) between subjects with one chronic illness and those with multiple chronic illnesses (p=0.42; Table 8). However, the indirect costs of illness varied with the type of illness, as cancers resulted in 45.1% lost productivity as compared with diabetes and hypertension that resulted in about 15% lost productivity. The monthly health expenditure of those who could satisfy their basic needs was statistically lower than that of those who could not (p=0.02; Table 8). Patients and families who spent less than 10% of their total monthly income on health were more likely to have their basic needs satisfied than those who spent more on health (p=0.001; Table 7).

**Table 7:** Respondents’ characteristics and proportion of monthly income spent on chronic illness

	Proportion of monthly income spent on health		Remarks
Number of chronic illness	< 10%	> 10%	
Single illness	87 (28.3%)	220 (71.7%)	p = 0.001*
Multiple illnesses	17(12.5%)	119(87.5%)	
Unsatisfied basic need	< 10%	> 10%	
Yes	16 (15.4%)	155 (45.7%)	p = 0.001*
No	88 (84.6%)	184 (54.3%)	
*p-value is significant (<0.05)			

**Table 8:** Costs of illness by illness type, number of illnesses, and ability to satisfy basic needs SD: standard deviation

	n=443	Mean	SD	p-value
Type of illness		Mean monthly direct costs ($)	SD ($)	
Cancer	14	489.07	499.32	-
Chronic kidney disease	7	299.98	323.08	-
Mental illness	48	249.65	687.96	-
Bone or joint problem	37	169.22	287.23	-
Chronic liver disease	23	162.17	318.91	-
Diabetes	142	159.76	392.00	-
Hypertension	289	104.89	288.94	-
Heart disease	32	76.58	81.74	-
Anemia	1	49.92	-	-
Asthma	6	42.33	33.87	-
Sickle cell disease	1	31.95	-	-
No of illnesses		Mean number of days (days)	SD (days)	
Single illness	307	5.0	10.11	0.42
Multiple illnesses	136	5.9	10.94	
Ability to satisfy basic needs		Mean direct costs ($)	SD ($)	
Yes	287	108.71	238.95	0.02*
No	156	191.07	508.77	
*p-value is significant (<0.05)				

Effect of employment on monthly income

Considering the average household monthly incomes and expenditures before and after the onset of illness by employment status, it is clear that those in paid employment had a higher mean monthly income both before and after the illness than those without a paid employment. However, the differences between the two groups were not statistically significant in both cases (before and after illness; p>0.05; Table 9). Also, when the change in monthly income as a result of the illness is considered, it appears that those in paid employment had a lesser change in monthly income, although the difference between the two groups, as seen in Table 9, was not statistically significant (p>0.05). Those who were in paid employment had more money available for spending on other essentials ($219.99±687.74) than those who did not have a paid employment ($180.30±561.51). Nonetheless, these averages were comparable between the two groups as the difference between them was not statistically significant (p>0.05). 

**Table 9:** Average household monthly incomes before and after illness onset by employment status SD: standard deviation

Average monthly income/expenditure	Paid employment (n=286)		Unpaid employment (n=157)		
	Mean ($)	SD ($)	Mean ($)	SD ($)	p-value
Household monthly income before the illness	362.94	694.87	286.46	379.66	0.20
Household monthly income since the illness started	349.82	595.46	260.06	391.40	0.09
Change in monthly income	13.12	230.59	26.39	161.09	0.52
Amount available to be spent on other things	219.99	687.74	180.30	561.51	0.08

## Discussion

In our sample of 443 respondents, approximately 65% of them were in paid employment while the remaining were either unemployed or retired. Being in a paid employment often implies additional benefits accruing to an employed person for most workers in Nigeria. In a way, a worker receives entitlements in forms of wages, bonuses, paid sick leaves, and sometimes employer-paid healthcare which may cushion the effects of treating any chronic illness within the household. First, those who were in paid employment experienced a lesser change in monthly income as a result of the disease ($13.12), as against those who were not in paid employment who experienced a relatively larger change in monthly income of $26.39 (p > 0.05). Secondly, those who had paid employment had a higher average monthly income ($362.94) relative to those who were not in paid employment ($286.46), which suggests that employed individuals have additional funds to cater for the costs of illness, and a relatively reduced burden of chronic disease on the family. Also, those who were in a paid employment had more money to spend on other essential things ($219.99) as against $180.30 for those who were not in a paid employment (Table 9). This difference between the two groups was not significant (p>0.05), but suggests that being in a paid employment can somewhat cushion the effects of chronic illnesses on households. However, in many developing countries where unemployment rates are very high and paid employment is not readily available to cushion the costs of chronic illnesses on families, the effects can be very devastating. 

While not all the respondents had to pay for admission or other specific forms of treatment as the requirements of managing each disease varied, almost all had to pay for drugs and investigations. It is expected that laboratory investigations and drugs are essential parts of any disease management which should be common to all the chronic illnesses studied [5]. However, the disease-specific needs may have accounted for the wide differences in the mean direct cost for each treatment parameter as some respondents, for instance, went through hospital admissions while others did not. Of the treatment parameters, drugs constituted the largest proportion of the direct costs to the respondents. It has been explained that direct costs include medical and non-medical costs but the proportions spent on these may be varied due to supply factors such as availability, accessibility, and user fee policy [8]. Some respondents may have to travel long distances because of the unavailability of specialist care. Particularly in Nigeria, as well as many other LMICs where chronic illnesses are mostly managed in tertiary healthcare faculties which are, largely few and mostly owned by the government; the cost of transportation becomes very significant. This forces many of the respondents to pay more for transport and, therefore, makes non-medical costs a larger proportion of direct costs for such subjects (as reflected in the data spread and huge standard deviations). It is also a reflection of the accessibility to needed care, implying that specialized services to manage chronic illnesses are not freely available, especially when patients have to travel far to access care.

The average household monthly incomes before and after illness onset were found to be $335.84 and $318.01 respectively, showing a mean drop of $17.82. This unexpectedly small mean change in monthly income despite the fact that some subjects were not able to work at all, or had an increased absenteeism from work, could have been due to the time frame as the duration of illness was as long as 30 years in some subjects. As a result of this, using the resulting change in monthly income as one of the economic effects of chronic illness is flawed [5, 11]. However, other studies have found a statistically significant fall in expected earnings as a result of chronic illness within five to six years [12].

With a mean direct cost of $137.72 and an average household monthly income of $318.01, the average proportion of the household income spent on health was 43.3%. It has been shown that a direct cost burden of illness greater than 10% is catastrophic [8, 13-15] even though some studies have used 40% as the benchmark for catastrophic health spending [16]. This means the average household should not spend more than $31.80 (10%) in order not to experience significant financial stresses. However, the majority of the households spent far beyond that. In this study, only 21.9% of the respondents spent less than 10% of their monthly income while about 43% spent more than 40% of monthly income on their illness. Unlike studies reporting that the majority of households incurred a low burden of direct illness costs because of access to free public healthcare for the regular treatment of chronic illnesses and inpatient care, this study reflects the deficiency of such protective measures [17, 18]. A large direct cost burden may also reflect the relatively low household income, as direct cost burden has an inverse relationship with the household income [9]. As seen in this study (Table 3), about 46% of the households earned less than the mean direct cost of treating their illness ($137.72).  

One way to determine the indirect cost of illness is to use the lost labor time due to the illness which translates to a reduced household capacity to earn income (especially at a time when it needs additional money to pay for treatment) [8]. The indirect costs of illness for both the patients and caregivers are 5.30 and 1.42 days respectively, implying that caregivers devoted an average of two days in a month to take care of their sick relatives. This is a widely used indicator of the effect of illness on the labor supply [5]. Converting these figures to a monetary value using the gross domestic product (GDP) per capita of Nigeria of $2,436 (at the time of study), the mean loss per subject was $503.92, annually, which is equivalent to 1.5 months’ average income as found in the study, while the caregiver lost an average of $134.56 annually- equivalent to approximately 50% of the average monthly wage [19-20]. This shows a significant economic impact even though this indicator fails to capture other indirect costs, including intangible costs that are difficult to estimate. Nonetheless, the huge loss of productivity and income can have significant implications on a country’s economic status, particularly if the prevalence of chronic illnesses continues to rise in LMICs as recent estimates have shown [2-3]. 

In response to chronic illness, the most common coping strategies used included borrowing (44.7%), health insurance (8.6%), and selling off of assets (5.0%). For those that borrowed money, the major sources were friends and families (together amounting to 73.7%). Russell [4] explains that friends and families constitute the most important source of social support, and this has been buttressed by these results. Other sources were from the bank, cooperative societies, and churches. Obtaining bank loans for use on health is difficult in the study environment. However, cooperative societies have taken the place of banks for a lot of people, making it easier to save and also obtain overdrafts when the need arises without having to provide significant collaterals. The fact that 44.7% of the respondents borrowed money in this study is in contrast to a Thai study which showed that only 15% borrowed money [21]. This may have been due to the low coverage of health insurance found in this study (8.6%) in contrast to 76% in Thailand [22]. This contrast between Nigeria and Thailand also points at the need for Nigeria, as well as other LMICs, to improve the penetration of and accessibility to health insurance. Many developed nations have well-established national health insurance systems which guarantee healthcare based on clinical need at affordable levies/premiums [18]. Total out of pocket cost of all illnesses in such environments is usually much less than 10% of the monthly household income at the point of care for most people [8]. This goes a long way in protecting the average household from the catastrophic and devastating effects that the treatment of a patient’s chronic illness has. 

About 85% of the respondents had caregivers, a finding that reflects the influence of social networks on the economic burden of chronic illness on households in our environment. According to Russell [4], social networks are important in the provision of viable coping strategies, the most vital component of this network being the immediate family. Therefore, the presence of a large proportion of caregivers may account for the additional sources of funds to cater for the direct costs of illness, especially in situations where the cost of treating the illness is more than the monthly income of the patient or household. Many of our respondents got additional funds for their illness, mostly from friends and family (74% altogether), further confirming the importance of caregivers as important providers of coping strategies. It also points at how many households have been coping with the lack of health insurance which has been recognized as a major intervention in minimizing the effects of chronic illness on the household [23]. 

It is important to note that the costs of illness vary with the type of disease. As presented in Table 8, patients with cancer spent significantly more ($489.07) than those with sickle cell disease or asthma, who spent much less. Illnesses such as chronic kidney disease, chronic liver disease, and diabetes resulted in relatively high direct costs. These are illnesses of high severity, which suggests that the more serious and longer-term the illness is, the more burden it places on the family. As Russell [8] has shown, it is likely that households who have to deal with huge costs of managing a disease will struggle or fail to cope with the costs, becoming impoverished or even failing to survive as a social unit. This has been depicted in Table 1, in which about 46% of the households earned less than the mean direct cost of treating chronic illness ($137.72) in this study. For these families to cope, they have to depend on the social resources at their disposal. This is in line with several other studies that have revealed the importance of social resources for households faced with illness costs beyond their budgets [24-26].

Considering the findings obtained and presented herein, it is clear that one patient's chronic illness can be catastrophic on his/her household in terms of the economic implications. Unfortunately, formal health insurance is lacking for many Nigerian households, making them bear the full brunt of these catastrophic effects. Although many of these households have devised other coping strategies, including borrowing from friends or family and selling of vital assets, the effects of managing the patient’s chronic illness make them lose vital productivity and income, and further pushes them into poverty. These huge economic effects that households bear have significant implications for health policy in Nigeria and other LMICs. Using the example of Thailand and many other countries that have successfully improved the coverage of health insurance, the economic implications of chronic illnesses on households can be significantly minimized with increased coverage of insurance-based financing systems. There is also the need to improve access to effective and specialized health care services that are responsive to the needs of people who have chronic illnesses. 

In addition, the findings of this study highlight the importance of health promotion and disease prevention to reduce both the direct and indirect costs of illness to households as well as reduce the cost of running national health services [27-28]. Strength lies in the use of established cost-of-illness methods for estimating economic implications of diseases. However, the major limitations encountered includes difficulty in assessing the monetary value of labor losses due to ill health over a long period of time due to inflation, difficulty in estimating the actual household monthly income (especially for non-salary earners who are not in paid employment), and the exploratory nature of the study that relied on self-reported income and expenditure with the possibility of recall biases. Hence, there is the need for further research on the household costs of illness, household responses, their implications for poverty eradication in LMICs, and how these impacts can be alleviated considering the effects of economic variables.

## Conclusions

There is a need to realize how devastating the economic effects of chronic illnesses are on the families of patients who suffer these illnesses, particularly in Nigeria, as well as other LMICs. It is evident that most families do not have formal support, having to manage the high and catastrophic cost burdens that chronic illnesses place on them. The lack of effective coping strategies also points at the need for policymakers to improve access to specialized care and increase coverage of formal health insurance so as to ameliorate the significant economic impacts that chronic illnesses have on entire households.

## Figures and Tables

**Figure 1:**
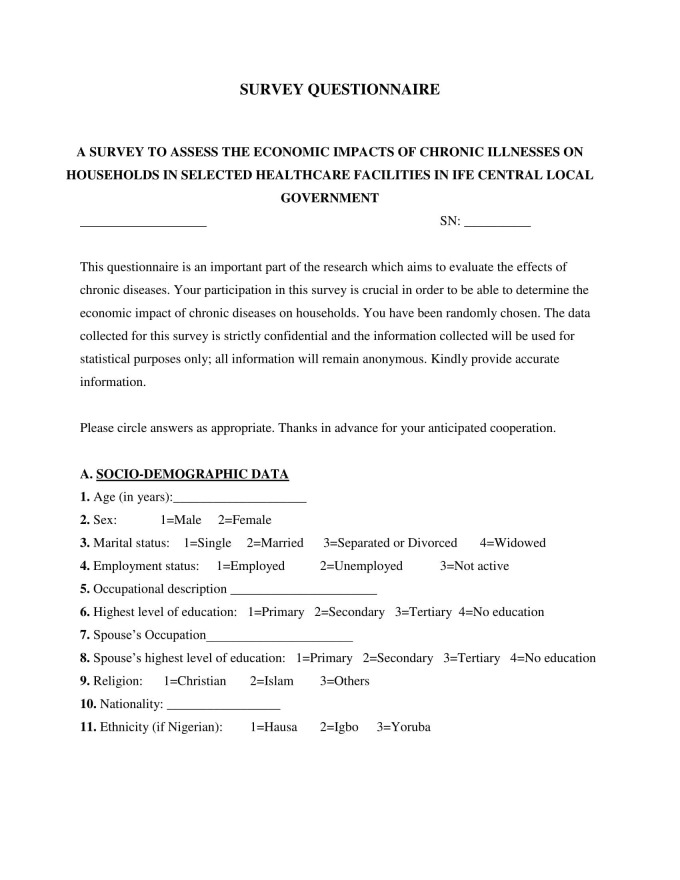
Survey questionnaire - Page 1

**Figure 2:**
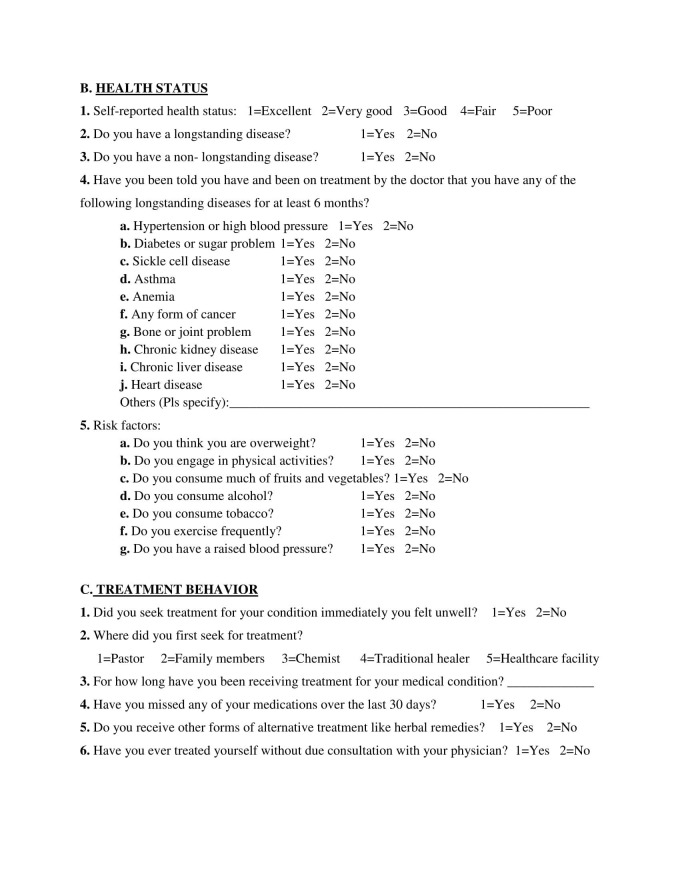
Survey questionnaire - Page 2

**Figure 3:**
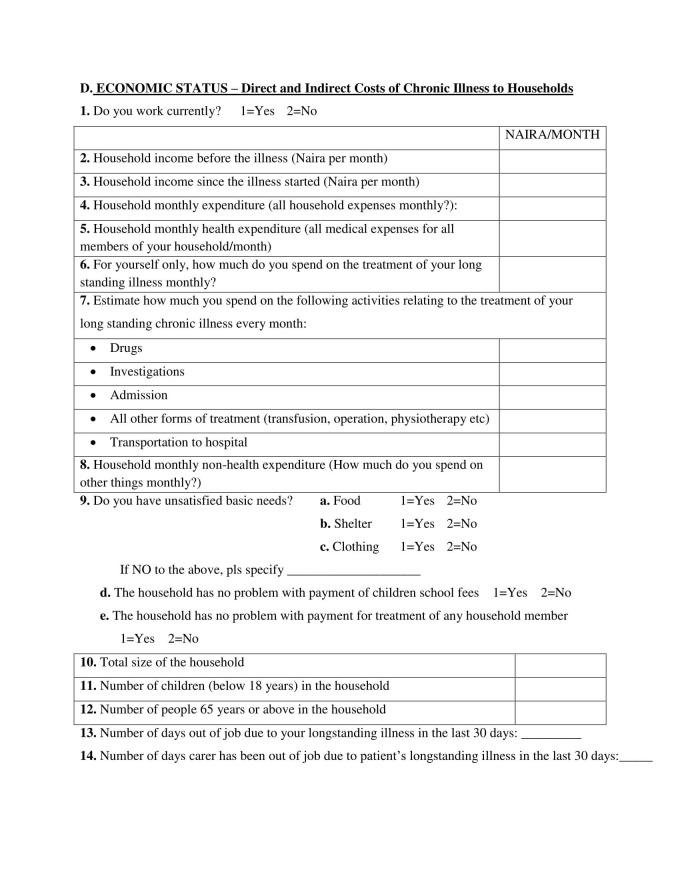
Survey questionnaire - Page 3

**Figure 4:**
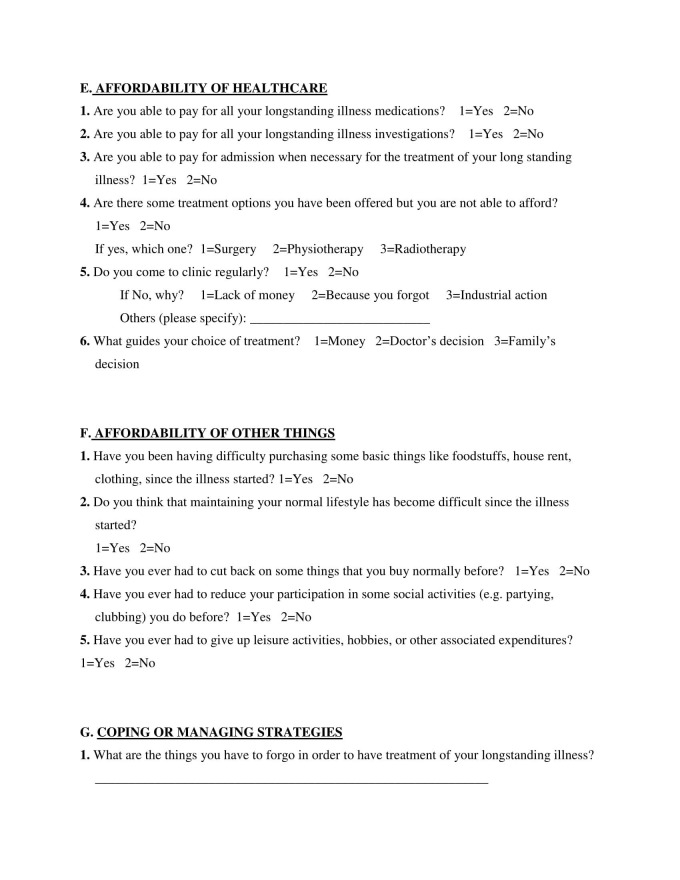
Survey questionnaire - Page 4

**Figure 5:**
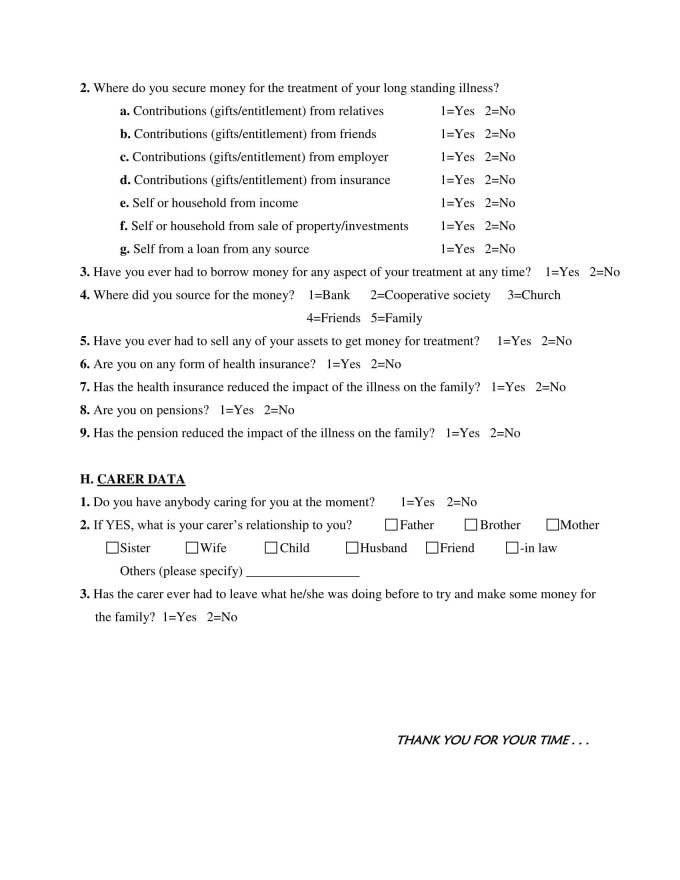
Survey questionnaire - Page 5
